# Tumor-To-Tumor Metastasis of Poorly Differentiated Gastric Carcinoma to Uterine Lipoleiomyoma

**DOI:** 10.1155/2015/352369

**Published:** 2015-11-25

**Authors:** Ryo Kiyokoba, Hiroshi Yagi, Hideaki Yahata, Yoshiaki Kawano, Eisuke Kaneki, Kaoru Okugawa, Kenzo Sonoda, Kiyoko Kato

**Affiliations:** Department of Obstetrics and Gynecology, Graduate School of Medical Sciences, Kyushu University, Fukuoka 812-0052, Japan

## Abstract

The rare phenomenon of tumor-to-tumor metastasis was first described in 1930. The donor neoplasm is most frequently lung or breast carcinoma, whereas intracranial meningiomas are reportedly the commonest recipient neoplasm. Here we report a case of metastasis from a primary gastric cancer to a uterine lipoleiomyoma. A 65-year-old woman presented with locally advanced gastric cancer with computed tomography (CT) evidence of peritoneal dissemination and a 9 cm pelvic mass. She underwent 16 courses of TS-1/cisplatin chemotherapy, which achieved significant tumor reduction. However, repeat CT and magnetic resonance imaging revealed a 9 cm diameter pelvic mass adjacent to the uterus. The mass was heterogeneously hyperintense on T1- and T2-weighted images with some low signal spots on fat-suppressed T1-weighted images, suggesting a benign ovarian tumor such as a mature cystic teratoma. After 3 months, pelvic CT revealed a 10 cm multilocular cystic mass that exhibited heterogeneous enhancement after intravenous contrast administration. A diagnostic laparotomy revealed a subserosal uterine tumor extending into the right broad ligament; total abdominal hysterectomy and bilateral salpingo-oophorectomy was performed. The uterine tumor showed histological features of lipoleiomyoma infiltrated by well- to moderately differentiated carcinoma cells that were similar to those of the gastric biopsy, supporting a diagnosis of metastatic gastric adenocarcinoma.

## 1. Introduction

Other than by direct invasion from adjacent pelvic organs, metastasis of extragenital cancers to the uterus is uncommon. In addition, metastases to uterine lipoleiomyomas, a rare variant of leiomyoma, are extremely rare. The rare phenomenon of metastases to histologically distinct tumors was first described in 1930 [1] and is known as tumor-to-tumor metastasis [2]. As for the recipient tumor, intracranial meningiomas are reportedly the commonest [2], whereas breast cancer is the commonest donor tumor [2]. Here we present a case of gastric cancer that metastasized to a uterine lipoleiomyoma.

## 2. Case Report

A 62-year-old woman, gravida 2 para 2, presented with abdominal pain. Gastrointestinal endoscopy showed superficial gastric erosions and histopathological examination of endoscopic biopsy specimens revealed poorly differentiated adenocarcinoma. Staging computed tomography (CT) scan demonstrated thickening of the gastric sinus wall, peritoneal dissemination, and 9 cm diameter solid pelvic mass. Based on these findings, the patient was diagnosed as having advanced (stage IV) gastric cancer and treated with TS-1/cisplatin (CDDP) chemotherapy. After 16 courses of TS-1/CDDP chemotherapy, gastroscopy showed complete resolution of the ulcerous lesion and CT demonstrated significant reduction in peritoneal dissemination. However, the pelvic tumor had not changed in size and had developed a cystic component. The patient was referred to our hospital for evaluation of the pelvic tumor.

**Figure 1:**
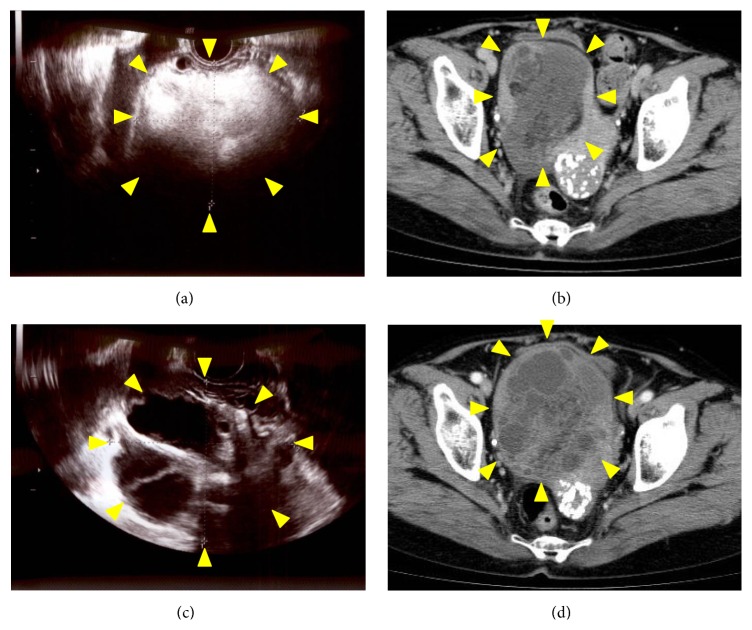
On referral to our institution, transvaginal ultrasonography (a) and computed tomography (CT) (b) demonstrated a 10 cm pelvic tumor. Three months later, transvaginal ultrasonography showed a multilocular tumor (c) that showed heterogeneous enhancement on contrast-enhanced CT (d). Arrowheads indicate the pelvic tumor.

**Figure 2:**
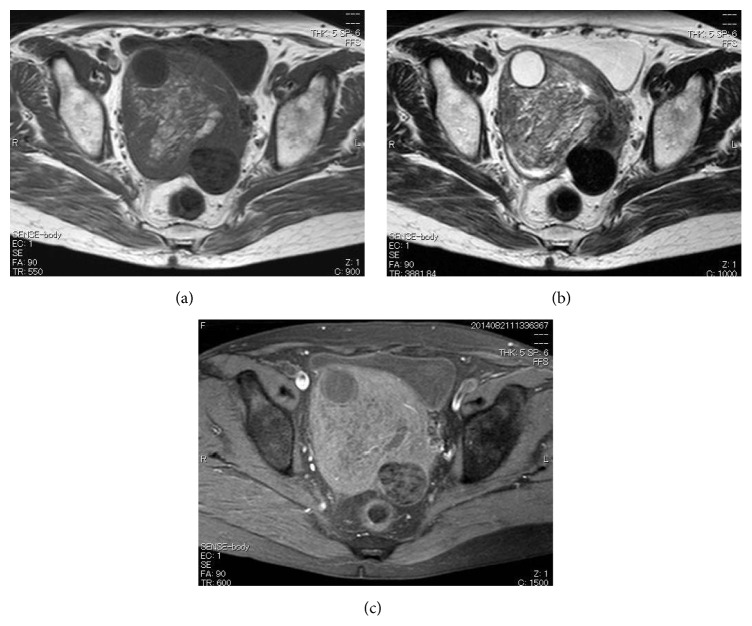
On referral to our institution, transverse magnetic resonance imaging sections through the pelvic tumor. (a) T1-, (b) T2-, and (c) fat-saturated T1-weighted images.

At the first visit to our hospital, the patient's general condition was good and her medical and family histories unremarkable. On pelvic examination, a firm, nontender, approximately 10 cm diameter mass was palpated in the right lower abdomen. Transvaginal ultrasonography and computed tomography (CT) revealed a 10 cm diameter pelvic mass (Figures 1(a) and 1(b)). Magnetic resonance imaging (MRI) demonstrated a tumor with mixed low- and high-signal intensities on both T1- and T2-weighted images (Figures 2(a) and 2(b)). Moreover, fatty elements were identified in the tumor on fat-saturated T1-weighted images (Figure 2(c)). Smear cytology of the endometrium was negative. Laboratory data, including serum concentrations of cancer antigens 125 and 19-9, carcinoembryonic antigen, and squamous cell carcinoma antigen, were within normal limits. We therefore suspected a benign right ovarian tumor, such as a mature cystic teratoma, and elected to follow the patient up closely without further treatment. Three months later, transvaginal ultrasonography demonstrated a multilocular cystic mass that differed markedly from that seen on referral to our hospital. Contrast-enhanced CT revealed a 10 cm sized multilocular cystic mass with heterogeneous enhancement (Figures 1(c) and 1(d)). However, the peritoneal dissemination was stable and no new metastatic lesions were detected. As malignancy, including primary or metastatic ovarian tumor could not be excluded by the imaging studies, diagnostic laparotomy was performed. Examination of the pelvic cavity revealed a subserosal uterine tumor extending into the right broad ligament and total abdominal hysterectomy and bilateral salpingo-oophorectomy was performed.

**Figure 3:**
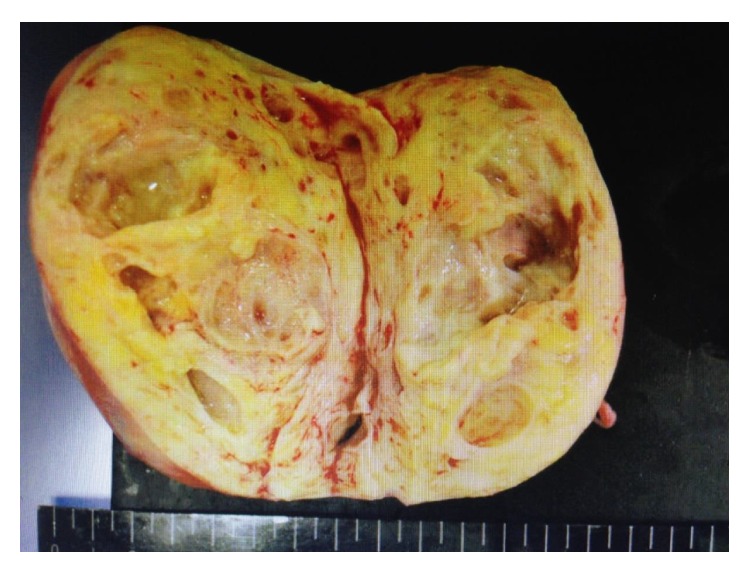
Cut surface of the operative specimen of the subserosal uterine tumor.

**Figure 4:**
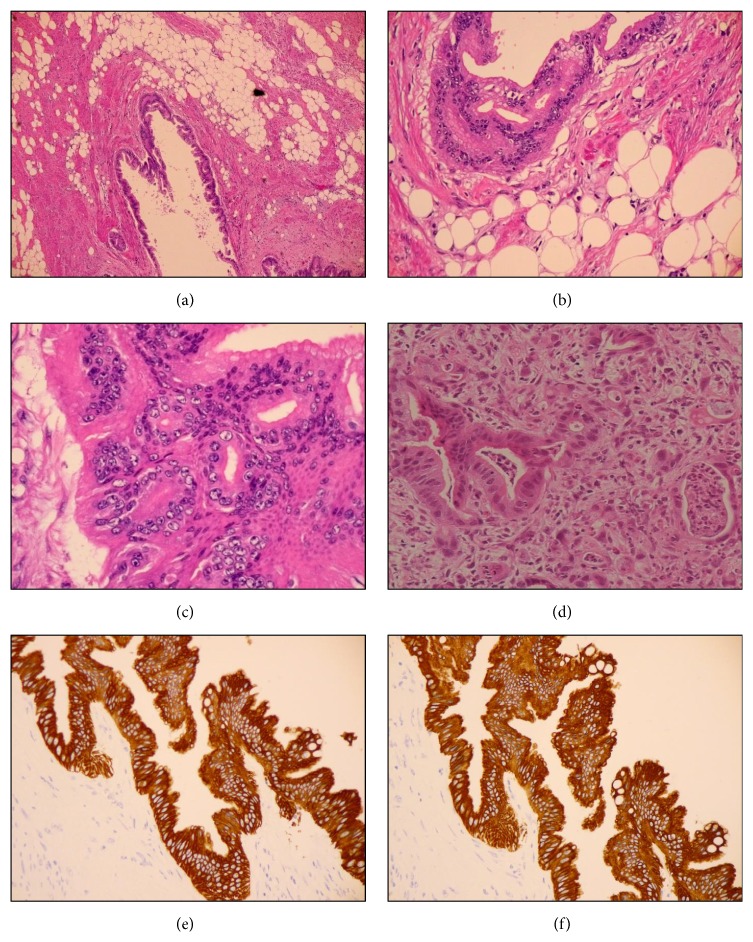
((a)–(c)) Photomicroscopy images showing lipoleiomyoma infiltrated with well- to moderately differentiated adenocarcinoma. (d) Biopsy specimen from the previous endoscopy showing poorly-differentiated adenocarcinoma. Stain, hematoxylin and eosin. ((e) and (f)) Representative immunohistochemistry for (e) CK7 and (f) CK20. Both CK7 and CK20 were positive in well- to moderately differentiated adenocarcinoma.

The subserosal uterine tumor measured 10 cm in diameter and had a yellow-white cut surface; the cysts contained yellow serous fluid (Figure 3). No areas of necrosis or hemorrhage were detected (Figure 3). The endometrium was atrophic. Both ovaries and fallopian tubes were macroscopically normal. On histological examination, the uterine tumor showed features of lipoleiomyoma with a mixture of mature fat and fascicles of muscle fibers (Figures 4(a) and 4(b)). It was infiltrated by well- to moderately differentiated carcinoma cells (Figures 4(a)–4(c)), which on immunohistochemical staining were found to be positive for cytokeratin (CK) 7, CK20, and CDX2, but negative for estrogen and progesterone receptors (Figures 4(e) and 4(f)). These features are compatible with metastatic adenocarcinoma of the previous gastric cancer, but not endometrial cancer infiltrating uterine myometrium (Figure 4(d)). Additionally, lymphatic permeation was detected in the body of the uterus. Peritoneal washing cytology analysis did not reveal any malignant cells.

The patient's postoperative course was uneventful, and she was discharged on the seventh day. The TS-1/CDDP chemotherapy was resumed and she remained alive with disseminated metastases 14 months after the surgery.

## 3. Discussion

Lipoleiomyoma of uterus is a rare variant of uterine leiomyoma, the incidence reportedly being 0.03% to 0.2% of all uterine tumors [3]. Histologically, these tumors are composed of benign smooth muscle cells and mature adipose tissue. Uterine lipoleiomyomas are common in obese menopausal women and characteristically asymptomatic. Their pathogenesis remains unclear because fat tissue is not native to the myometrium. The two main possibilities are adipose metaplasia of smooth muscle cells and a multipotential Müllerian cell origin [4]. On sonography, they appear as hyperechoic masses partly encased in a hypoechoic rind. CT shows encapsulated, heterogeneous, predominantly fatty tumors [3, 4]. MRI confirms the uterine location of the tumor and its lipomatous nature [4]. The differential diagnosis includes mature ovarian teratoma, benign pelvic lipoma, liposarcoma, extra-adrenal myelolipoma, lipoblastic lymphadenopathy, and retroperitoneal cystic hamartoma. However, uterine lipoleiomyomas are often diagnosed preoperatively as uterine leiomyomas or mature ovarian teratomas.

Other than by direct invasion from adjacent pelvic organs, metastasis of extragenital cancers to the uterus is uncommon [5]. Moreover, metastases to uterine leiomyomas are extremely rare; only 19 cases have been reported so far [2, 5–11]. In these 19 cases, the primary tumors originated in the breast (12), stomach (three), pancreas (two), and lung and bladder (one case each). In six of these cases, the metastases were confined to leiomyomas. Cancer cells metastasize to the uterine corpus, including to leiomyomas, by lymphatic or hematogenous pathways. Most uterine metastases are secondary to local lymphatic spread from preceding ovarian metastases; however, when the ovaries are not affected, as in the present case, the metastases are hematogenous [5].

The phenomenon of tumor-to-tumor metastasis was first described in 1930 [1]. To date, 104 cases have been reported [2, 5–11]. The donor neoplasms originated in the breast (65 cases), lung (12), kidney (seven), prostate (five), stomach (four), colon (three), skin and pancreas (two cases each), and uterine cervix, ovary, bladder, and salivary gland (one case each). Any benign or malignant tumor can be a recipient; however, meningiomas have been implicated as the most common neoplasm to harbor metastases. The second most frequent recipient neoplasm is reportedly uterine leiomyoma; 19 cases, including 18 of leiomyoma and one of lipoleiomyoma, have been reported [5, 7–11]. The preference of cancer cells for various metastatic sites depends on cross-talk between cancer cells (the “seeds”) and specific organ environments (the “soil”) [12]. Some clinical and biological characteristics of recipient tumors, such as slow growth rate, hypervascularity, and high collagen and lipid content, may play prominent roles in metastatic seeding.

## References

[1] Fried B. M. (1930). Metastatic inoculation of a meningioma by cancer cells from a bronchogenic carcinoma. *The American Journal of Pathology*.

[2] Moody P., Murtagh K., Piduru S., Brem S., Murtagh R., Rojiani A. M. (2012). Tumor-to-tumor metastasis: pathology and neuroimaging considerations. *International Journal of Clinical and Experimental Pathology*.

[3] Prieto A., Crespo C., Pardo A., Docal I., Calzada J., Alonso P. (2000). Uterine lipoleiomyomas: US and CT findings. *Abdominal Imaging*.

[4] Aizenstein R., Wilbur A. C., Aizenstein S. (1990). CT and MRI of uterine lipoleiomyoma. *Gynecologic Oncology*.

[5] Kumar N. B., Hart W. R. (1982). Metastases to the uterine corpus from extragenital cancers. A clinicopathologic study of 63 cases. *Cancer*.

[6] Jeong B., Shim J.-Y., Kim C. J., Won H.-S., Lee P. R., Kim A. (2014). Massive perivillous fibrin deposition in the placenta and uterine metastasis of gastric adenocarcinoma during pregnancy. *Journal of Obstetrics and Gynaecology Research*.

[7] Işçi H., Güdücü N., Basgul A. Y., Aydinli K., Calay Z., Dünder I. (2011). Lobular carcinoma of the breast metastasizing to leiomyoma in a patient under letrozole treatment. *European Journal of Gynaecological Oncology*.

[8] Kondo N. I., Yoshida S., Kajiyama H., Nagasaka T., Uematsu T. (2009). Metastasis of breast cancer to a uterine leiomyoma. *Breast Cancer*.

[9] Liebmann R. D., Jones K. D., Hamid R., Lapsley M. (1998). Fortuitous diagnosis in a uterine leiomyoma of metastatic lobular carcinoma of the breast. *Histopathology*.

[10] Uner A., Tiras M. B., Kilic D., Dursun A., Dilek U. (2003). Uterine lipoleiomyoma containing metastatic breast carcinoma: a case with two unusual pathologies. *European Journal of Obstetrics Gynecology and Reproductive Biology*.

[11] Le Bouedec G., de Latour M., Kauffmann P., Reynaud P., Fonck Y., Dauplat J. (1993). Uterine metastases originating from breast cancer. Apropos of 12 cases. *Archives d'Anatomie et de Cytologie Pathologiques*.

[12] Fidler I. J. (2003). The pathogenesis of cancer metastasis: the ‘seed and soil’ hypothesis revisited. *Nature Reviews Cancer*.

